# Cortical Mapping of Mismatch Negativity with Deviance Detection Property in Rat

**DOI:** 10.1371/journal.pone.0082663

**Published:** 2013-12-12

**Authors:** Tomoyo Isoguchi Shiramatsu, Ryohei Kanzaki, Hirokazu Takahashi

**Affiliations:** 1 Department of Mechano-Informatics, Graduate School of Information Science and Technology, The University of Tokyo, Tokyo, Japan; 2 Research Center for Advanced Science and Technology, The University of Tokyo, Tokyo, Japan; 3 Research Fellow of Japan Society for the Promotion of Science, Tokyo, Japan; 4 Precursory Research for Embryonic Science and Technology, Japan Science and Technology Agency, Saitama, Japan; Penn State University, United States of America

## Abstract

Mismatch Negativity (MMN) is an *N*-methyl-d-aspartic acid (NMDA)-mediated, negative deflection in human auditory evoked potentials in response to a cognitively discriminable change. MMN-like responses have been extensively investigated in animal models, but the existence of MMN equivalent is still controversial. In this study, we aimed to investigate how closely the putative MMN (MMNp) in rats exhibited the comparable properties of human MMN. We used a surface microelectrode array with a grid of 10×7 recording sites within an area of 4.5×3.0 mm to densely map evoked potentials in the auditory cortex of anesthetized rats under the oddball paradigm. Firstly, like human MMN, deviant stimuli elicited negative deflections in auditory evoked potentials following the positive middle-latency response, termed P1. Secondly, MMNp exhibited deviance-detecting property, which could not be explained by simple stimulus specific adaptation (SSA). Thirdly, this MMNp occurred focally in the auditory cortex, including both the core and belt regions, while P1 activation focus was obtained in the core region, indicating that both P1 and MMNp are generated in the auditory cortex, yet the sources of these signals do not completely overlap. Fourthly, MMNp significantly decreased after the application of AP5 (D-(-)-2-amino-5-phosphonopentanoic acid), an antagonist at NMDA receptors. In stark contrast, AP5 affected neither P1 amplitude nor SSA of P1. These results provide compelling evidence that the MMNp we have examined in rats is functionally comparable to human MMN. The present work will stimulate translational research into MMN, which may help bridge the gap between electroencephalography (EEG)/magnetoencephalography (MEG) studies in humans and electrophysiological studies in animals.

## Introduction

Mismatch Negativity (MMN) refers to a negative deflection in human auditory evoked potentials (AEP) in response to a cognitively discriminable change [1]–[4]. In the auditory oddball paradigm, particular changes such as frequency and intensity of stimulus sounds can elicit the MMN response, regardless of attention or consciousness [5]–[7]. Importantly, MMN appeared for complex stimuli such as categorical changes of chord consonance and grammatical error [8], [9], which are not explained by a mere effect of stimulus-specific adaptation (SSA). Thus, MMN is considered to be an automatic mechanism of detection for ‘deviants’ based on short term memory or “the primitive intelligence” of the auditory cortex [10]. Additionally, MMN is subject to change with learning and experience, suggesting that MMN is involved in higher-order brain functions [11]–[18]. MMN has proven useful in clinical diagnosis: for example, MMN is reduced in schizophrenia and predicts recovery from coma [19]–[27].

MMN-like responses have been extensively investigated in animal models [28]–[32], but the existence of MMN equivalent is still debated because, in some recent studies, the mismatch responses in animals were best explained by SSA rather than deviant detection [33]–[35]. In addition, this issue is more controversial in rodent models because of considerable variation in data among studies, possibly due to variation of reference electrode position or anesthetic agents [36]–[45]. Thus, comprehensive experiments are still required to conclude how closely the putative MMN (MMNp) in animals meets the general characteristics of MMN in humans. Specifically in rodents, characterization of MMNp may have important implications because rodents are useful as experimental models in the field of auditory neuroscience.

In the present study, in order to test whether MMNp in rodents exhibits comparable properties to human MMN, we attempted to densely map AEP in the auditory cortex of rats using a surface microelectrode array and to spatio-temporally characterize mismatch responses in an oddball paradigm. We examined how closely MMNp in rats exhibits the following 4 properties. First, MMN is elicited by deviant auditory stimuli in an oddball paradigm shortly after the earliest and largest component of AEP, termed P1. In several animal models, MMN-like responses appeared at 50–150-ms post-stimulus latency [28]–[32], [36]–[44], [46]–[50], which is shorter than human MMN latency (100–300 ms) [51]–[53]. Second, MMN exhibits deviance detection property; in animal studies, this possibility can be addressed by the “many standards control” paradigm, which is designed to remove SSA component from traditional MMN in an oddball paradigm [33], [34], [45], [54]. Third, the auditory cortex is known to be the origin of MMN. Electroencephalography (EEG)/magnetoencephalography (MEG) studies indicate that both MMN and P1 are generated in the auditory cortex, yet the sources of these signals do not completely overlap [8], [55]–[58]. MMN is often estimated to originate in the higher-order auditory field rather than in the primary auditory cortex. This is also supported by dense mapping of MMN in a cat model [31]. Fourth, MMN is mediated by *N*-methyl-d-aspartic acid (NMDA) receptors; an antagonist of NMDA receptors consistently disrupts both human MMN and MMN-like responses in animal models [29], [44], [49], [59], but does not affect SSA of P1 [34]. Our comprehensive experiments demonstrated, for the first time, that the mismatch responses of rodents in an oddball paradigm exhibited all the above-mentioned properties of MMN.

## Materials and Methods

This study was carried out in strict accordance with “Guiding Principles for the Care and Use of Animals in the Field of Physiological Science” published by the Japanese Physiological Society. The experimental protocol was approved by the Committee on the Ethics of Animal Experiments at the Research Center for Advanced Science and Technology, the University of Tokyo (Permit Number: RAC07110). All surgery was performed under isoflurane anesthesia, and every effort was made to minimize suffering. After the experiments, animals were euthanized with an overdose of pentobarbital sodium (160 mg/kg, i.p.).

For statistical tests in data analyses, t-test was used in pair-wise comparisons and Games-Howell test or t-test with Bonferroni correction was used in multiple comparisons.

### Animal preparation

Eighteen Wistar rats, at postnatal week 8–10, with a body weight of 210–290 g, were used in total. Six rats were used to test whether MMNp exhibited the deviance detecting property. Other twelve rats were used to investigate whether MMNp was mediated through NMDA receptors. Rats were anesthetized with isoflurane in conjunction with air (3% at induction and 1–2% for maintenance), and were held in place with a custom-made head-holding device. Atropine sulfate (0.1 mg/kg) was administered subcutaneously at the beginning and at the end of the surgery to reduce the viscosity of bronchial secretions. A heating blanket was used to maintain body temperature at approximately 37°C. The skin incision at the beginning of the surgery was made under local anesthesia of xylocaine (0.3–0.5 ml). A needle electrode was subcutaneously inserted into the right forepaw, and used as a ground. A small craniotomy was made near the bregma landmark to embed a 0.5-mm thick integrated circuit socket as a reference electrode, with an electrical contact to the dura mater. The right temporal muscle, cranium, and dura overlying the auditory cortex were surgically removed, and the exposed cortical surface was perfused with saline in order to prevent desiccation. Cisternal cerebrospinal fluid drainage was performed in order to minimize cerebral edema. The right eardrum, i.e., ipsilateral to the exposed cortex, was ruptured and waxed to ensure unilateral sound inputs from the ear contralateral to the exposed cortex. Respiratory rate, heart rate, and hind-paw withdrawal reflexes were monitored throughout the experiment in order to maintain an adequate anesthetic level as stably as possible.

### Auditory evoked potential mapping

A surface microelectrode array was used to map AEPs over the auditory cortex. The microelectrode array was made on a flexible polyimide substrate to conform to the curvature of cortical surface, with a grid of 10×7 recording sites within an area of 4.5×3.0 mm. Each recording site was 50×50 µm, and the electrode impedance was approximately 400 kΩ under 1-kHz, 0.1-V sinusoidal waves. Of particular interest in the present study were P1 and the subsequent MMNp. The dense mapping using surface microelectrode array revealed the spatial distributions of these waves over the entire auditory cortex.

A speaker (10TH800, Matsushita Electric Industrial Co. Ltd., Japan) was positioned 10 cm from the left ear, i.e., contralateral to the exposed cortex. Test stimuli were calibrated at the pinna with a 1/4-inch microphone (Brüel & Kjær, 4939) and spectrum analyzer (Ono Sokki Co., Ltd., CF-5210). The stimulus level is presented in dB SPL (sound pressure level in decibels with respect to 20 µPa).

AEPs were recorded from the surface microelectrode array mounted on the exposed auditory cortex. Neural signals were obtained with an amplification gain of 1,000, digital filter bandpass of 0.3–500 Hz, and sampling frequency of 1 kHz (Cyberkinetics Inc.; Cerebus Data Acquisition System).

The spatial distribution of click-evoked responses was first mapped on the cortical surface in order to identify the location of the auditory cortex. Clicks were presented 60 times to obtain the grand average of click-evoked responses. A click was a monophasic positive wave with duration of 0.5 ms and frequency range of 1–100 kHz. The microelectrode array was placed so that the lower and posterior ends of array approximately matched the ventral and posterior boarders of tone responsive area, respectively. Then, the recording area of 4.5×3.0 mm covered the entire auditory cortex, including the core and belt regions [60]. The primary and anterior auditory fields (A1 and AAF) were included in the core region, while other tone responsive areas, including the ventral and suprarhinal auditory fields (VAF and SRAF), were considered as the belt region. The core region exhibited larger P1 with 2–3 ms earlier latency than the belt region when a click or high-intensity tones were presented [60]. The recording area was then putatively divided into 3 regions: the core region, including the 25 recording sites showing the largest response amplitudes at P1; the belt region, comprising recording sites located ventral and posterior to the core region; and the non-auditory region, which encompassed the remaining recording sites. The area covered by 25 recording sites was 4.8 mm^2^, which approximates to the combined area of A1 and AAF (4–6 mm^2^) according to previous microelectrode mapping studies [61]–[65] (see Discussion for further validation).

**Figure 1:**
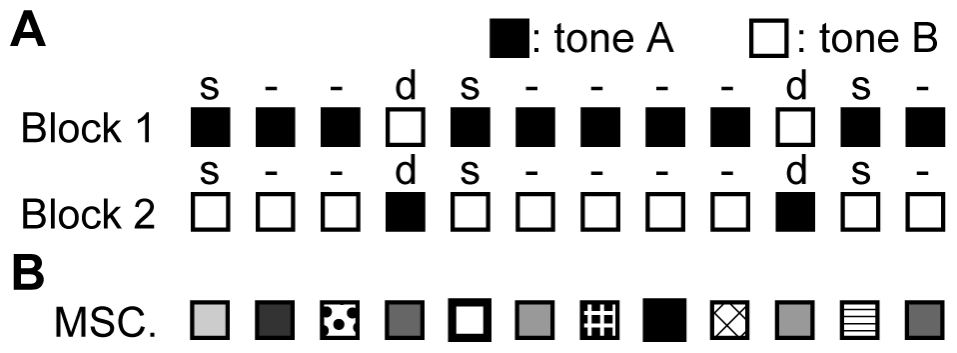
Test paradigm. (A) Oddball paradigm. Tones (A and B) used for standard (s) and deviant (d) were alternated in Block 1 and Block 2. Deviant tones were randomly delivered with an appearance probability of 10%. (B) Many standards control paradigm (MSC). Tones with 10 different frequencies were presented randomly. Note that the appearance probability (10%) of each tone was identical to that of the deviants in the oddball paradigm.

Test stimuli were tone bursts with a 60-dB SPL plateau, and 100-ms duration including 5-ms rise/fall times. The inter-stimulus interval was fixed at 600 ms. AEPs were recorded during an oddball paradigm, as shown in Fig. 1A. The test stimulus sequences consisted of 2 tones with differing frequencies, i.e., tone A and tone B, serving as either a standard or deviant. Standards were presented with a probability of 90%, and deviants with a probability of 10%. In each block, 540 standards and 60 deviants were randomly presented, and the grand average of standard AEP and deviant AEP were obtained. As summarized in Table 1, four test pairs of tone A and tone B were used in the experiment. With these test frequencies that cover the entire audibility range of rats, we were able to investigate whether MMNp had a tonotopic distribution as well as P1 and whether MMNp and SSA depended on the frequency difference. The frequency difference was defined as

**Table 1:** Sound frequency of tone pairs used in the experiments.

	tone A: *f_A_* (Hz)	tone B: *f_B_* (Hz)	ΔF (small/large)
pair 1	1,000	1,260	0.232 (small)
pair 2	6,349	8,000	0.232 (small)
pair 3	40,317	50,000	0.216 (small)
pair 4	6,349	16,000	0.958 (large)

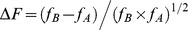
) is indicated in the rightmost column, and categorized into either small or large condition. In the first and second blocks, standard and deviant tones were alternated in order to derive the MMNp of either tone A or tone B by subtracting the deviant-evoked response from the standard-evoked response. Hence, a total of 8 stimulus conditions were tested. The frequency difference (



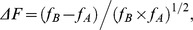
where *f_A_* and *f_B_* were test frequencies of tone A and tone B, respectively; ΔF was categorized into large and small conditions as shown in Table 1. In the first and second blocks, standard and deviant tones were alternated in order to derive the MMNp of either tone A or tone B by subtracting the deviant-evoked response from the standard-evoked response. Hence, a total of eight stimulus conditions were tested. The order of the blocks and the stimulus conditions was randomized. To quantify how SSA depended on ΔF, normalized SSA indices (SI) were derived as



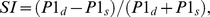
where P1_s_ and P1_d_ were standard P1 and deviant P1, respectively [66].

In six rats, in order to exclude the possibility that MMNp is the mere effect of SSA and to test whether MMNp exhibited the deviance detection properties, AEPs were additionally investigated in the “many standards control” paradigm (Fig. 1B). In this control paradigm, tone bursts with 10 different frequencies were presented randomly. The test frequencies were 1,000, 1,260, 3,175, 6,349, 8,000, 12,000, 16,000, 27,000 40,317 and 50,000 Hz, seven of which were used in the oddball paradigm (Table 1). The appearance probability of each test frequency was identical to that of deviants; yet, because the stimulus sequence had no abrupt change unlike the oddball paradigm, MMNp was not expected [33], [34], [45], [54].

### Administration of NMDA receptor antagonist

To investigate whether MMNp recorded during the oddball protocol is mediated by NMDA receptors, as is the case with human MMN, AEPs were also measured following the administration of AP5 (D-(-)-2-amino-5-phosphonopentanoic acid) directly onto the surface of the auditory cortex. In the first session, AEPs were measured immediately after the surgery. In the second session, AEPs were measured after a gel sheet of 1% (10 g/*l*) agarose containing 100 µM AP5 had been placed onto the cortical surface for 15 min (*n* = 6). In a control group (*n* = 6), AEPs in the second session were measured after the 15 min placement of a gel sheet without AP5. The control group eliminated the possibility of effects being due to the agarose gel itself, or to the gradual deterioration of cortical activity between the first and second sessions. Precise re-positioning of the microelectrode array was possible using vessel patterns as positional references, so that AEP maps were obtained from an almost identical location in the first and second sessions [60].

## Results

**Figure 2:**
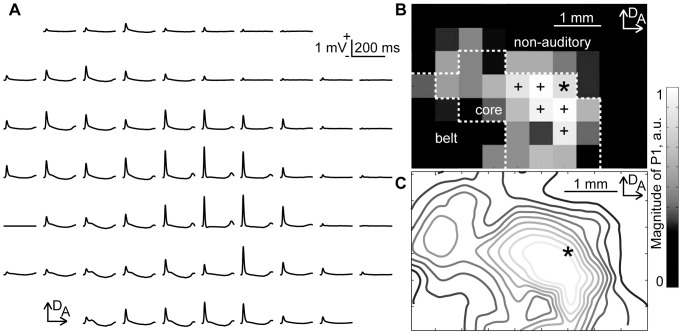
AEP map elicited by clicks. (A) Representative mapping of AEP waveforms. AEP was measured simultaneously with 64 recording sites. Each AEP waveform is approximately aligned in the spatial coordinates of the recording sites of the surface microelectrode array. The grand averages of 60 recordings are shown. (B) Spatial distribution of the click-evoked P1. The gray level at each grid corresponds to P1 amplitude measured at each electrode in the array. Recording sites producing the top 10% of P1 amplitudes are denoted by the markings (‘+’ and ‘*’). In this activation focus, the most anterior-dorsal site, indicated by the asterisk, is the positional reference used to pool data across animals. The figure also shows the delineation of the test regions: core, belt and non-auditory regions. (C) Contours of P1 distribution with cubic interpolation. Abbreviations: A, anterior; D, dorsal.

Figure 2A shows the representative cortical mapping of click-elicited AEPs. In these grand-averaged click-evoked responses, P1 waves were quantified as the maximum amplitude within 50 ms from the onset of stimulus. Figures 2B and 2C show the P1 amplitude at each recording site and a contour map of the P1 distribution, with cubic interpolation. This P1 distribution was used to pool data across subjects and determine the core, belt and non-auditory regions, according to the following procedure. The P1 amplitude at each recording site was normalized with respect to the maximum P1 among all sites for each animal. Then, a positional reference to superimpose individual AEP maps was determined as follows. First, the top 10% of recording sites in terms of P1 amplitude were extracted (‘+’ and ‘*’ in Fig. 2B). In this activation focus, the most anterior-dorsal site was used as the positional reference (‘*’ in Fig. 2B and 2C). This positional reference was appropriate for superimposing both A1 and AAF, which extended along the anterior-to-posterior and dorsal-to-ventral axes, respectively. The distributions of click-evoked P1 amplitude were superimposed across all rats. Then, based on the superimposed distribution of P1 amplitudes, the recording area was putatively divided into the core, belt, and non-auditory regions, as shown in Fig. 2B (See Materials and Methods).

**Figure 3:**
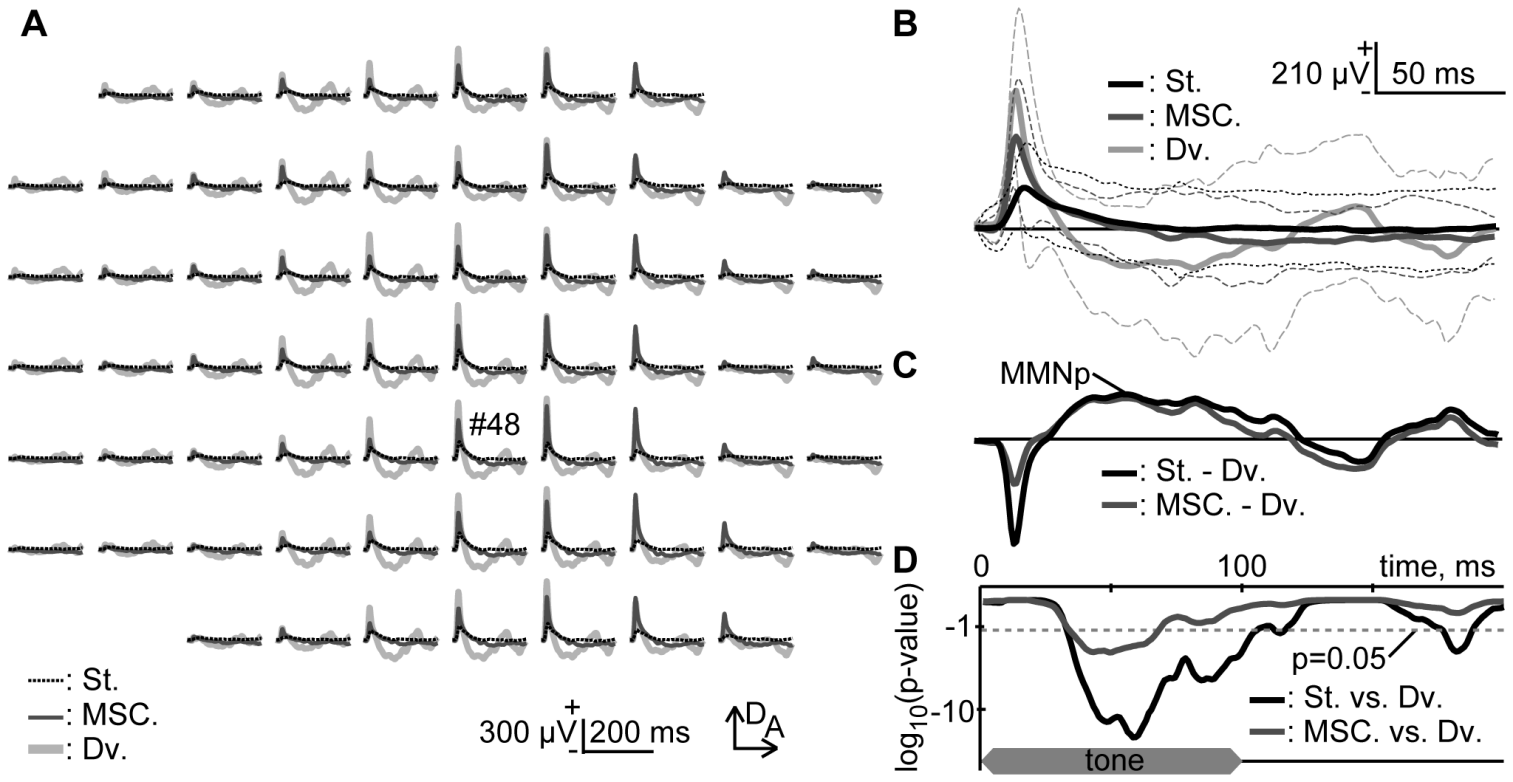
Pure-tone-evoked putative Mismatch Negativity (MMNp). (A) Representative mapping of AEP. These responses were obtained from 40,317 Hz tones under indicated conditions: St. (broken black) and Dv. (light gray), standard and deviant in the oddball paradigm; MSC. (dark gray), many standards control. In the oddball paradigm, a pair of 40,317 Hz and 50,000 Hz tones was used. (B) AEPs from an indicated recording site (#48). The mean and s.d. are given. (C) MMNp. MMNp was defined as the subtraction of deviant AEP from standard AEP (black). Difference wave between deviant AEP and many-standards-control AEP was also shown for comparison (gray). (D) Significance level under a null hypothesis that deviant AEPs (*n* = 60) are larger than standard AEPs (*n* = 540, black) or many-standards-control AEPs (*n* = 60, gray) at a given post-stimulus latency time (one-sided t-test with Bonferroni correction for 200 comparisons). The ordinate indicates log10 of the significance level. Broken line indicates p = 0.05. The time course of stimulus presentation is indicated at the bottom of the inset.

Figure 3A shows representative mappings of 40,317-Hz tone in the oddball and many standards control paradigms. In the oddball paradigm, a pair of 40,317 Hz and 50,000 Hz tones was used for test stimuli. As shown in Fig. 3B, representative AEPs exhibited P1 in all the conditions tested, while a negative deflection that followed P1 appeared distinctly only in the deviant responses. This deflection was defined as MMNp by subtracting the deviant AEP from the standard AEP, as shown in Fig. 3C. For comparison, the deflection was also quantified by subtracting the deviant AEP from the many-standards-control AEP, resulting in a nearly identical waveform to MMNp. Figure 3D statistically confirms that the negative deflections in deviants were significantly larger than those in controls and standards (one-sided t-test with Bonferroni correction for 200 comparisons, p<0.05).

**Figure 4:**
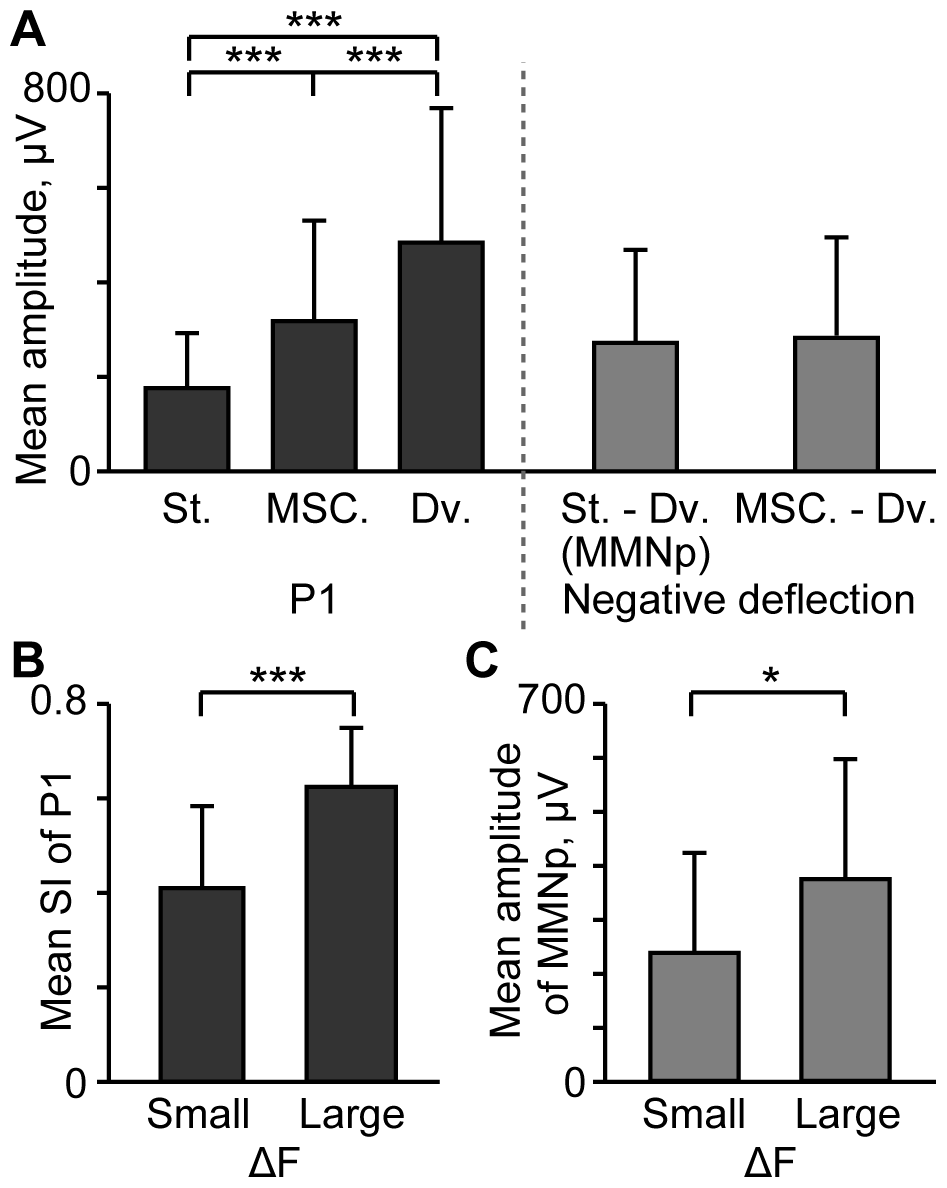
Amplitude of AEP components in oddball and many-standards-control paradigms. (A) P1 and negative deflection that follows are quantified in indicated conditions: St. and Dv., standard and deviant in the oddball paradigm; MSC., many standards control; St. – Dv., subtraction of deviant AEP from standard AEP (MMNp); MSC. – Dv., subtraction of deviant AEP from many-standards-control AEP. The mean and standard deviation are given (*n* = 6 (animal)×8 (stimulus condition)). Asterisks indicate statistical significance: ***, p<0.001 (two-sided t-test with Bonferroni correction for 3 comparisons). (B) SI depending on ΔF. Asterisk indicates statistical significance: ***, p<0.001 (two-sided t-test) (C) MMNp depending on ΔF. Asterisk indicates statistical significance: *, p<0.05 (two-sided t-test).

Figure 4A shows the peak amplitude (mean ± s.d.) of P1 and negative deflections in indicated conditions (*n* = 6 (animal)×8 (stimulus condition)). The largest P1 was obtained from deviant responses, followed in order by the many-standards-control responses and standard responses (two-sided t-test with Bonferroni correction for 3 comparisons, p<0.001). The P1 latencies were 25.0±6.3 ms for the standards, 22.7±4.5 ms for the deviants, and 22.7±4.1 ms in the many standards control paradigm; statistically, the standard P1 was significantly later than either the deviant or many-standards-control P1 (one-sided t-test with Bonferroni correction for 3 comparisons, p<0.05). These results indicate that P1 exhibited SSA. On the other hand, the negative deflection did not differ whether deviant AEP was subtracted from standard AEP or many-standards-control AEP (paired t-test, p>0.1), excluding the possibility that SSA accounted for MMNp. The peak latency of MMNp was 81.6±28.1 ms.

To investigate whether SSA and MMNp depended on ΔF, Figures 4B and 4C compare SI and the amplitude of MMNp between the small ΔF (*n* = 6 (animal)×6 (stimulus condition)) and large ΔF conditions (*n* = 6 (animal)×2 (stimulus condition)). Consequently, the large ΔF conditions resulted in larger SI (two-sided t-test, p<0.001) and larger MMNp (p<0.05) than the small ΔF conditions.

**Figure 5:**
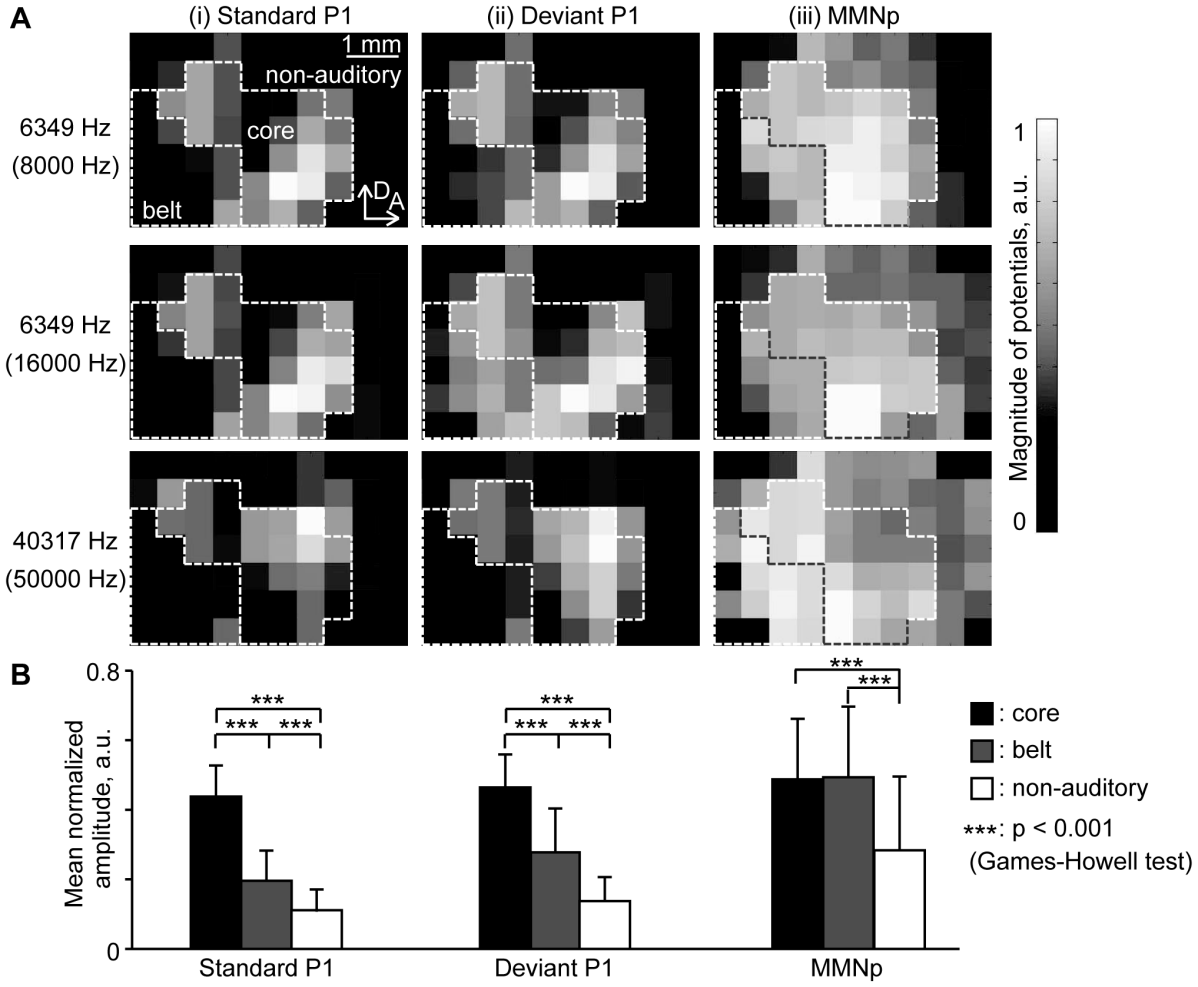
Spatial distribution of P1 and MMNp. (A) Spatial distributions of individual data: (i) P1 for standard stimuli; (ii) P1 for deviant stimuli; and (iii) MMNp. These AEPs were obtained from 6,349-Hz pure tones paired with either 8,000 Hz tones or 16,000 Hz tones, and 40,317 Hz tones paired with 50,000 Hz tones. (B) Regional differences in response amplitudes among the core, belt, and non-auditory regions. The mean and s.d. of region-specific amplitude are given (*n* = 12 (animal)×8 (stimulus condition)). Asterisks indicate statistical significance: ***, p<0.001 (Games-Howell test).

Figure 5A shows how the spatial distributions of standard P1 (i), deviant P1 (ii), and MMNp (iii) depended on test tone frequency: 6,349 Hz tone paired with 8,000 Hz tone (pair 2 in Table 1); 6,349 Hz tone paired with 16,000 Hz tone (pair 4); 40,317 Hz tone paired with 50,000 Hz tone (pair 3). For both standard and deviant tones, P1 had activation foci in the core region, which were spatially dependent on test frequency. The P1 spatial distributions were consistent with the tonotopic map of the auditory cortex: the low frequency tone of 6,349 Hz elicited activation at the posterior region in A1 and at the anterio-ventral region in AAF, resulting in a mirror image; in contrast, the high frequency tone of 40,317 Hz elicited activation at the anterior region in A1 and at the posterio-dorsal region in AAF, resulting in focal activation at the boarder of A1 and AAF, i.e., center of the core region. As compared to P1, MMNp tended to be spread more widely over the auditory cortex, including both the core and belt regions, and exhibited less clear foci, with no apparent tonotopic structure.

To visualize regional differences, Fig. 5B quantifies the normalized amplitudes of P1 and MMNp in the core, belt, and non-auditory regions, separately: in each AEP map, i.e., under each stimulus condition in each animal, the normalized response amplitudes within test regions were averaged, and these region-specific amplitudes were averaged across animals and conditions (*n* = 12 (animal)×8 (stimulus condition)). Region-specific P1 of both standard and deviant responses were largest in the core regions, followed by the belt and non-auditory regions (Games-Howell test, p<0.001). Region-specific MMNp, on the other hand, was significantly larger in the auditory cortex than in the non-auditory regions (Games-Howell test, p<0.001), yet did not significantly differ between the core and belt regions (p>0.1). These results suggest that both P1 and MMNp have their origins in the auditory cortex, yet the origins are not identical with each other.

**Figure 6:**
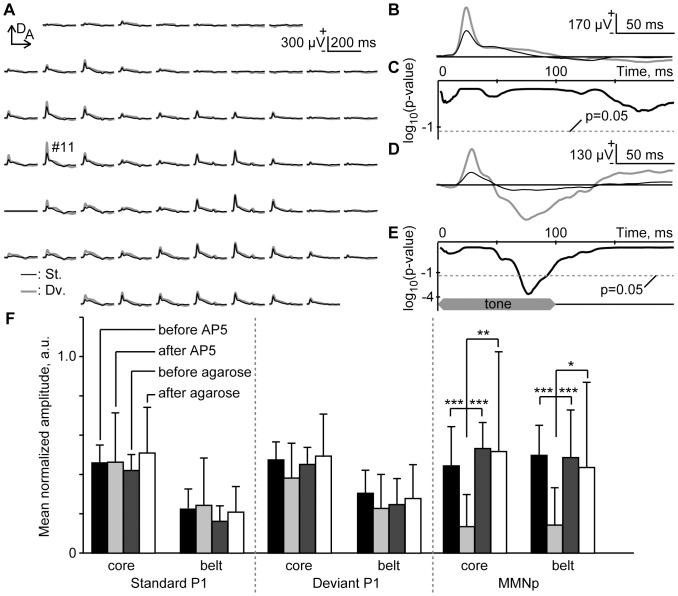
Pharmacological effects of NMDA antagonist (AP5) on AEP. (A) Representative mapping in an oddball paradigm of responses to 6,349 Hz and 8,000 Hz tones. These responses were obtained from 8,000 Hz tones. (B) Representative standard and deviant AEPs recorded at ch #11 after AP5 administration. The grand averages of standard (black) and deviant (gray) AEPs are shown. (C) Significance level under a null hypothesis that amplitudes of deviant AEPs are larger than those of standard AEPs at a given post-stimulus latency (one-sided t-test with Bonferroni correction for 200 comparisons). (D and E) Representative AEPs and significance level in the control group. (F) Normalized amplitude of standard P1, deviant P1 and MMNp in pooled data. Response amplitudes were compared before and after the placing of the agarose gel. The agarose contained AP5 in the test group, but not in the control group. The regional difference, i.e., between the core and belt regions, was also investigated. The mean and standard deviation of the normalized amplitude are shown (n = 6 (animal)×8 (stimulus condition)). Asterisks indicate statistical significance: *, p<0.05; **, p<0.01; ***, p<0.001 (Games-Howell test).

Lastly, Fig. 6A shows the mapping of standard- and deviant-evoked responses when AP5, an antagonist of the NMDA receptor, was administered. As shown in the representative traces (Fig. 6B), standard- and deviant-evoked responses in this experiment were almost identical, such that AP5 had little effect on P1, but eliminated MMNp (Fig. 6C). In the control group, on the other hand, MMNp was clearly evident, as shown in Figs. 6D and 6E.

At the largest MMNp after AP5 administration, the amplitude and latency were 90.0±57.3 µV and 113.8±29.5 ms, respectively, while 178.4±123.9 µV and 97.7±27.1 ms in the control treatment. At the largest P1 after AP5 administration, the amplitude and latency were 451.3±224.6 µV and 20.1±2.2 ms, respectively, for standards, while 671.7±388.0 µV and 20.2±4.0 ms for deviants; in the control treatment, the amplitude and latency were 371.0±160.5 µV and 21.5±3.7 ms for standards, while 585.6±315.1 µV and 20.5±2.9 ms for deviants. In both AP5 and control treatment groups, deviants elicited significantly larger P1 than standards (two-sided t-test, p<0.001), indicating that SSA was effective in P1 independently of NMDA receptor.

To quantify the spatial effect of AP5, the response amplitudes of either P1 or MMNp were normalized with respect to the maximum amplitude among all recording sites in the first session, i.e., without AP5. For each animal, the spatial average of the normalized amplitudes was obtained in the core and belt regions separately, and was averaged across animals (*n* = 6 (animal)×8 (stimulus condition)), as shown in Fig. 6F. Consequently, no significant effects of AP5 were found on either standard- or deviant-evoked P1. On the other hand, MMNp was selectively diminished after AP5 administration, as compared to the 3 other conditions, i.e., before AP5 administration and before/after control treatment (Games-Howell test, p<0.05). Both in P1 and MMNp, no region specific difference was found in the AP5 effects (Games-Howell test, p>0.1).

## Discussion

In this study, a surface microelectrode array was used to map AEPs in the auditory cortex of anesthetized rats. In an oddball paradigm, a mismatch response with negative polarity was consistently elicited, which we refer to as MMNp throughout this study. This MMNp exhibited the 4 general properties of MMN in humans. Firstly, in the oddball paradigm, MMNp followed P1 with a post-stimulus latency of 50–150 ms. Secondly, the many standards control paradigm demonstrated that SSA was observed in P1 but not in MMNp, indicating the deviance detection property of MMNp. Consistent with previous studies, both SSA and MMNp were enhanced with the increase of frequency difference between standard and deviant tones [66]. Thirdly, MMNp was spatially distributed within the auditory cortex, yet the distribution differed from that of P1: the activation focus of P1 was observed in the core regions, i.e., A1 and AAF, while MMNp was recorded not only from the core regions, but also from the belt regions. Fourthly, pharmacological experiments demonstrated that NMDA receptors mediate MMNp, but not SSA of P1. To our knowledge, this is the first report demonstrating that the mismatch response in the rodent simultaneously meets multiple requirements of MMN, providing compelling evidence that MMNp in the rodent is functionally comparable to MMN in human.

### Morphological characteristics of MMNp

The latency of MMNp in the present study, i.e., 50–150 ms, was nearly identical to that reported in other animal studies, where mismatch responses were characterized as negative deflections [41], [42]. In contrast, MMN latency in humans is typically 100–300 ms [51]–[53], which is longer than the MMNp latency in this study. Generally, MMN-like responses appears earlier in animal models than in humans [28]–[32], [36]–[44], [46]–[50]. Empirically, the latency increases with the size of the brain [67]. Thus, the difference in latency between human and animal models does not contradict the hypothesis that MMNp in this study is an equivalent of human MMN.

A number of previous studies reported that mismatch responses in rats appeared as a positive deflection [36]–[45]; however, a negative deflection was consistently observed in our experiments. Different placement of the recording electrodes may cause this discrepancy. For example, the polarity of MMN in humans reversed when the reference electrode was moved from the nose to the occipital area [36], [68]. In our study, a reference electrode was placed near the bregma landmark, while other studies have placed a reference electrode on the cerebellum [36], [39]. We avoided placing a reference electrode on the cerebellum because oddball stimuli were likely to activate the cerebellum as well [69], [70]. Additionally, anesthetic agents may have some profound effects on cortical responses. Urethane anesthesia may result in positive, or no MMN-like responses [36], [39], [40], [43]. Other studies have reported findings consistent with our results; negative MMN-like responses has been reported when fentanyl and medetomidine and/or isoflurane were used for anesthesia [42]. Anesthetic agents may also alter morphology of AEP: First, the P1 latencies were extended; second, negative component following P1, termed N1, was likely absent when the concentration of isoflurane was 1.25% or higher [71].

### Deviance-detecting property of MMNp

Traditionally, MMN has been characterized in oddball paradigms, where repeating stimuli cause attenuation of neural responses, i.e., SSA. Thus, the ‘traditional’ MMN may be dominated by SSA as well as by deviance-detecting properties, or ‘genuine’ MMN [54]. Recent studies have argued that MMN-like responses in animal models are different from human MMN because they could be explained by SSA, rather than by a deviance-detecting property [33]. To address this issue, the “many standards control” paradigm has been designed to remove SSA and examine the deviance-detecting property [33], [34], [45], [54].

In the many standards control paradigm, tones with 10 different frequencies were presented randomly. These frequencies ranged widely from 1 kHz to 50 kHz in order to eliminate SSA as effectively as possible [35]. As a result, P1 amplitude was larger in the many standards control than in the standard responses, demonstrating that SSA was reduced. The subsequent component, on the other hand, did not change in the many standards control, indicating that SSA is not the neural substrate of MMNp.

Furthermore, although the appearance probability of each tone in the many standards control was identical to that of deviant tone in the oddball paradigm, negative deflection following P1 was observed only in the oddball paradigm, but not in the many standards control. This indicates that the context of repeating standards as well as rareness of stimulus, i.e., the property of deviance, is required to elicit MMNp. Thus, MMNp is characterized as having a deviance-detecting property.

### Methodological consideration

To the best of our knowledge, this is the first study that has densely mapped MMNp in the rat auditory cortex and attempted to characterize region-specific properties of MMNp. In the present experiments, however, it was still difficult to precisely delineate the core and belt cortices of an individual subject because the spatial resolution of our surface microelectrode array (500 µm) is not sufficiently fine and LFP spreads with a spatial constant of 500 µm by nature [60], [72]. We have therefore adopted our empirical criteria to putatively delineate the core and belt regions once data were pooled across subjects. Our criteria to delineate the core region consisted of 3 steps. First, P1 elicited by click stimulus and high-intensity tones were twice larger in the core than in the belt cortex, allowing approximate localization of core region [60]. Second, the size of core region was considered: in the spatial map of P1, top 25 sites (corresponding to 4.8 mm^2^) were included in the core, because the estimate size of core region was 4–6 mm^2^ on the basis of tonotopic maps in a number of existing single/multi-unit studies [61]–[65]. Third, the remaining regions ventral and dorsal to the putative core region were regarded as the belt and non-auditory areas, respectively. This criterion is justified because we placed the microelectrode array so that the lower and posterior ends of array approximately matched the ventral and posterior boarders of tone responsive area, respectively. Although there were some auditory fields in the dorsal region of core, these fields were negligibly small in terms of contribution to AEP. Our criteria may cause mislabeling at a few sites by nature, but we believe that such errors are not severe enough to undermine the general validity of region specific properties of AEP characterized here.

### Spatial pattern of MMNp

In our mapping, we found that P1 and MMNp had different spatial distributions; the focal activation of P1 was in the core regions, while MMNp was recorded both in the core and belt regions. This result is consistent with previous MEG and EEG studies in humans, reporting that the origin of MMNp is slightly different from that of P1, and is often estimated to be in higher-order auditory areas than the origin of middle latency responses [8], [55]–[58]. The core cortex sends dense feedback projections to the peripheral auditory nuclei, but very sparse projections to the limbic and higher cognitive systems, while the belt cortex sends substantial projections to these brain regions [73]–[84]. In addition, long-term emotional memories are stored in the belt regions, but not in the core [85]. Thus, the involvement of the belt region in MMNp supports evidence from a number of human studies indicating that MMN is associated with higher order functions such as attention, learning, language and experience [11]–[18]. The involvement of NMDA receptors also supports the notion that MMN is associated with these functions [29], [44], [49], [59], [86].

Our results were partially inconsistent with previous dense mapping of the cat auditory cortex, which demonstrated that P1 was distributed over the primary auditory cortex, while the mismatch response was distributed over the secondary, but not the primary, auditory cortex. A potential cause of this discrepancy is interspecies differences in the thalamocortical pathway. In general, the primary auditory fields in cats, and the core regions in rats receive projections mainly from the ventral division of the medial geniculate body (MGB), while the secondary auditory field in cats, and the belt regions in rats receive projections from the dorsal and medial division of the MGB [73]–[79], [82], [84]. These 2 pathways are far more clearly segregated in cats than in rats [74]. If the medial and dorsal divisions of the MGB are crucial to the generation of MMN, such a difference in projections may result in a different spatial distribution of MMNp responses.

Our experiments provide compelling evidence that the MMNp we have investigated in rats is functionally comparable to human MMN. This finding in rodents may have significant implication in higher animals, in which MMNp is highly likely conserved during evolution, and stimulates further animal studies to investigate neural mechanisms of MMN-related clinical findings [19]–[27]. Furthermore, we have demonstrated that our surface microelectrode mapping technique is able to probe the spatial distribution of MMNp within the auditory cortex. These results will stimulate translational research into MMN, which may help to bridge the gap between EEG/MEG studies in humans, and electrophysiological studies in animals. For example, the spatial distribution of MMN was dependent on how a test tone sequence changes, either in frequency or intensity [87]–[90], with different tones possibly recruiting different population of neurons [91]. Furthermore, human studies suggest that the origin of MMN is dependent on more general stimulus properties such as pure tones, chords and melodies [8], [92]. Such stimulus-dependent MMN distributions are a possible indication of the spatial segregation of functions within the auditory cortex. Our experimental setup will significantly contribute to the resolution of these questions.
